# dbSMR: a novel resource of genome-wide SNPs affecting microRNA mediated regulation

**DOI:** 10.1186/1471-2105-10-108

**Published:** 2009-04-16

**Authors:** Manoj Hariharan, Vinod Scaria, Samir K Brahmachari

**Affiliations:** 1 G. N. Ramachandran Knowledge Center for Genome Informatics, Institute of Genomics and Integrative Biology (CSIR), Mall Road, Delhi, India

## Abstract

**Background:**

MicroRNAs (miRNAs) regulate several biological processes through post-transcriptional gene silencing. The efficiency of binding of miRNAs to target transcripts depends on the sequence as well as intramolecular structure of the transcript. Single Nucleotide Polymorphisms (SNPs) can contribute to alterations in the structure of regions flanking them, thereby influencing the accessibility for miRNA binding.

**Description:**

The entire human genome was analyzed for SNPs in and around predicted miRNA target sites. Polymorphisms within 200 nucleotides that could alter the intramolecular structure at the target site, thereby altering regulation were annotated. Collated information was ported in a MySQL database with a user-friendly interface accessible through the URL: .

**Conclusion:**

The database has a user-friendly interface where the information can be queried using either the gene name, microRNA name, polymorphism ID or transcript ID. Combination queries using 'AND' or 'OR' is also possible along with specifying the degree of change of intramolecular bonding with and without the polymorphism. Such a resource would enable researchers address questions like the role of regulatory SNPs in the 3' UTRs and population specific regulatory modulations in the context of microRNA targets.

## Background

Interaction of microRNAs (miRNAs) to specific sites in the transcripts of several human genes evidently, has profound effects on various biological processes like development, differentiation, proliferation, apoptosis, metabolism, host-pathogen interactions and cancer [1,2]. These ~17–25 nucleotide long molecules generally bind to the 3' untranslated regions (UTRs) of certain transcripts harboring complementary sites, thereby reducing its translational ability. Over 500 human miRNAs have been identified in the human genome, each of them having the potential to bind to hundreds of transcripts. The miRNAs form a complex with other proteins called miRNA-Protein complex (miRNP) or the miRNA Induced Silencing Complex (miRISC). This complex is known to interact with target sites with incomplete complementarity [3]. Several experiments have demonstrated that bases 2–7 from the 5' end of the miRNA are required to be exactly complementary to the sequence at the target site to form a 'seed' binding and a few mismatches of 3–5 bulged loops can be tolerated [4,5]. Another set of experiments demonstrate that a seed match is not mandatory if there is a compensatory pairing towards the 3' end of the miRNA in the bound complex that is sufficient to obtain an optimum free energy for the bound complex [6].

Variation of structure at the target site has been identified as another key factor that determines the interaction of miRNA to the target site. Long range interactions between bases in the RNA result in complex structures like pseudo-knots while there are short range interactions which mostly lead to stem-loop structures. While composition of the bases and the length of the stem region make some of these structures particularly stable, other conformations like the presence of internal loops, multi-branch loops or bulges could destabilize these structures. Conceivably, the target site of a particular miRNA might not always be open and accessible for the miRISC to interact with the site. It has been established that the miRNPs can effectively bind to target sites which do not have a highly structured conformation in comparison to a structurally stable target site [7]. The presence of stable structures 70 nucleotides (nt) flanking the respective target sites hindered hsa-miR-1 from downregulating thymosin β4 and Igf1 while the same miRNA could regulate the levels of Hand2 [8]. In another study, the sequence composition was altered to force a structural variation in order to confirm the accessibility preference of miRNA [9]. Based on these principles, others and we (unpublished web server) have implemented second generation of target prediction servers, which incorporate the accessibility of miRNAs to target site as another factor [10-13].

Several cases of dysregulation due to polymorphisms at the miRNA binding site have been reported. It was noted that the 3' UTR of SLITRK1 gene, a candidate of Tourette's syndrome, harboured a G-to-A polymorphism which stabilized the interaction of hsa-miR-189 since a A:U pairing is stronger that the G:U wobble; to facilitate collating of such SNPs that occur at the miRNA target site, a database called Patrocles had been developed [14]. Quantitative Trait Loci (QTL) mapping in sheep identified a gene GDF8 accounting for muscular dystrophy. This gene contained a G-to-A substitution in the 3' UTR that created a more stable site for two miRNAs miR-1 and miR-206. A three-fold reduction in GDF8 was observed [15]. A genome wide study has established that though SNPs at miRNA binding site are rare, few of them are positively selected in certain population [16]. Another such study of SNPs in miRNA binding sites of all human transcripts established that very few SNPs occur in the miRNA binding motifs and that aberrant allele frequencies were found in cancer ESTs [17]. Another example is the A-to-C polymorphism (rs5186) which disrupts the A:U pairing and consequently, the binding of hsa-miR-155 to the AGTR1 gene, possibly leading to hypertension [18]. A C-to-T polymorphism 14 nt downstream of the miR-24 target site on DHFR gene resulted in degradation of the target transcript [19].

**Figure 1:**
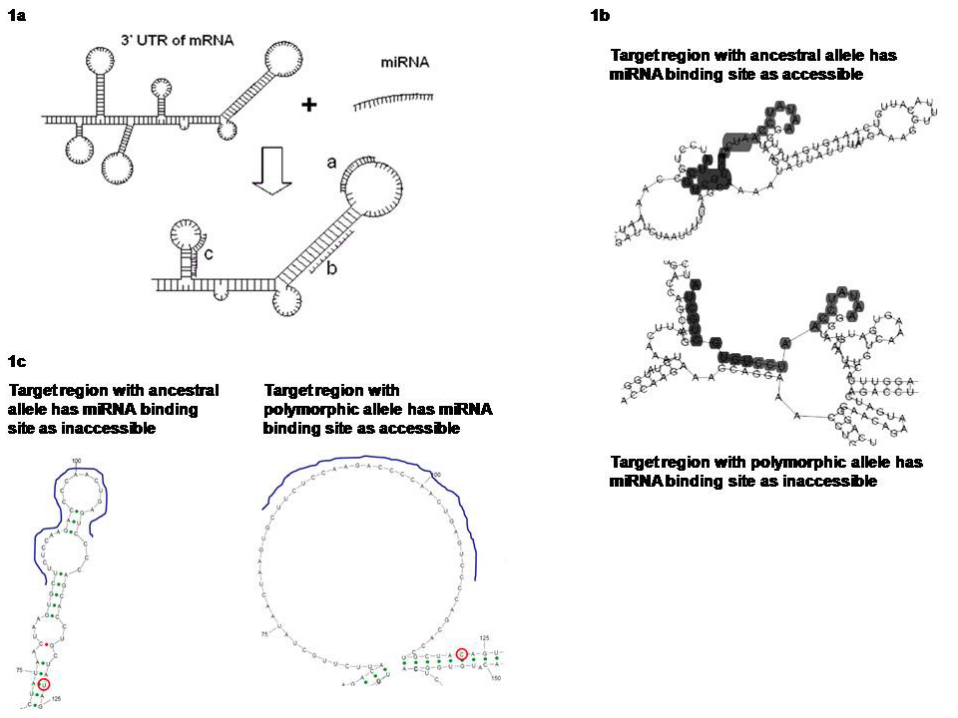
**miRNA-mRNA interaction model**. **a**: Case a: miRNA binding site in loop region of mRNA (Favorable), Case b: miRNA binding site in stem region of mRNA (Unfavorable), Case c: miRNA binding site in stem-loop junction (Sequence Dependent). **b**: The validated binding site for miR-15a/miR-16 in the 3' UTR of BCL2 was found to be accessible with the wild type allele, but the A-to-G polymorphism alters the intramolecular structure at the target site which could prove to be altering the accessibility of the miRNA to bind. The shaded region indicates miRNA binding site. **c**: The validated binding site for hsa-miR-24 in the 3' UTR of DHFR gene with 'U' allele 14 bp downstream is structured and hence, inaccessible for miRNA binding while the 'C' allele makes the target site totally unstructured thereby allowing miRNA binding.

Based on the experimental evidence mentioned above, it can be surmised that not miRNA binding at the target site is influenced not only by sequence changes within the target site, but also those hundreds of bases away, if they influence the secondary structure at the target site. Polymorphisms, either at the target site or around the target site, have the potential to alter the base-pairing patterns which in turn would determine the accessibility of the miRNA at the target site. A highly structured region (due to intramolecular bonds formed within the bases in the 3' UTR) would be inaccessible for miRNAs since the energy required to break the existing bonds would be insufficiently offset by formation of new bonds with an external molecule, the miRNA which is 17–25 bases long. Further, the large activation energy involved in destabilizing the mRNA secondary structure would render interactions within the secondary structure forming regions kinetically non-feasible even when thermodynamically viable. This would be especially pronounced for those miRNAs do not bind to the target sites with complete complementarity. Conversely, miRNA binding to those regions which are either not continuously bound for a long stretch or which are wholly unbound to any base in the 3' UTR would be energetically favored (Figure 1a).

## Construction and Content

Targets to all human miRNAs, obtained from miRBase database v9 [20], were predicted in the 3' UTR sequences downloaded from the Ensembl database [21] using the BioMart feature. Currently available miRNA target prediction tools are associated with a large number of false positives and as an alternative, results which agree between two or three algorithms would be better to identify the most probable miRNA-target pairs [22]. We used three software – miRanda, RNAHybrid and TargetScan to detect the miRNA target pairs [23-25]. Only those miRNA-target pairs were selected which were predicted to bind to the same target site by all the three software.

We further analyzed the subset of SNPs that are located within 200 nt of the predicted miRNA-target pairs, by extracting two sets of sequences, one with the wild type allele and other with the polymorphic allele at the 201^st ^position of this stretch. Further, we computationally determine the presence of secondary structures using the RNAFold program for both the sequences [26]. Computational prediction of RNA secondary structure has limited accuracy in predicting long-range interactions, complex structures like pseudo-knots, structures of long sequences (>1 kb). We focused on sequence stretches of 400 nt for two reasons: (a) the long-range interactions might be overcome by the steric hindrance caused by miRNPs; and (b) presently available secondary structure prediction tools have an optimum efficiency for sequence of length 400–700 bases [27].

We then extracted the structural information of the 3' UTR at the site where the miRNA is known to bind in the case of wild and polymorphic sequence. The bases involved in intramolecular base-pairing is denoted by an 'X' while a '-' denotes an unbound base. We calculate the change in number of bases changing its structural conformation and the ratio of the number of bases changing the intramolecular structure at the target site to the total number of bases binding to the miRNA gives a degree of change in the overall structural variation. The degree of change in the intramolecular bonds formed is an indication of the affect of the SNP in the intramolecular structure change at the particular target site.

**Figure 2:**
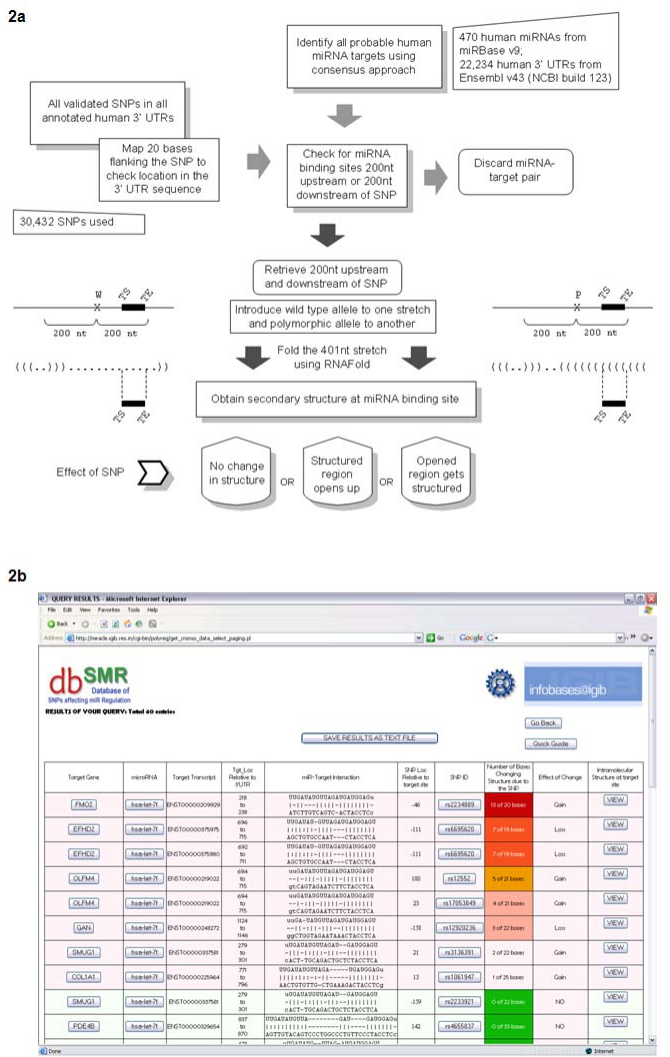
**Summary of methodology and result interpretation**. **a**: The work-flow of the current analysis: In the pictorial representation, the W and P represents the wild and polymorphic alleles on the 201^st ^position, TS and TE represent the target site start and end. The secondary structure obtained for the 400 nt stretch is in the form of brackets (representing structured bases) and dots (representing unbound bases). b: Screen-shot of query result with miRNA hsa-let-7f. **b**: A result display of the database. The columns give the information as target gene name, miRNA target the transcript, the transcript ID, the location of miRNA binding with respect to the 3' UTR of the transcript, the binding modality, the location of SNP with respect to the target site, the SNP ID, the number of bases changing intramolecular conformation, the effect of the change (as gain or loss), and a link-out to pictorial depiction of the structural change.

Mathematically,



A greater number of structured bases in the target site of the polymorphic stretch would imply loss of a legitimate miRNA binding site due to the polymorphism, while a lesser number of structured bases in the polymorphic sequence implies a gain of an target site. A similar procedure is followed to detect the intramolecular structures formed at the sites where miRNAs are validated experimentally to bind to the UTRs of the few genes from the TarBase database [28]. Figure 2a gives a detailed workflow of the present study.

The data is ported in a MySQL database that is accessible through a user-friendly web-interface through codes written in CGI-PERL. Various query options exists by which users obtain information regarding the miRNA, the transcript, corresponding gene, the binding site in the UTR, the SNP around the miRNA binding site, the distance of separation of SNP from the target site and a visual representation of the intramolecular structure at the miRNA binding site in the 3' UTR. Depending on the decreasing significance in variation of the intramolecular structure, the details are colored in various shades of red. In cases where the SNP does not alter the structure, the information is colored in green (Figure 1b). The users also have the option of saving the results of their query as a tab-separated text file. Table 1 gives a summary of findings of the analysis and the data ported.

**Table 1:** Overview of predicted effect of SNPs on miRNA target binding

Intramolecular Structure at miRNA binding region	Category	DSNP	TSNP	USNP	TOTAL
Significant Change	Loss	139	68	108	315
Significant Change	Gain	62	55	90	207
Moderate Change	Loss	1230	638	1215	3083
Moderate Change	Gain	1187	726	1147	3060
No Change	-	15573	1396	13968	30937
Total	-	18191	2883	16528	37602

The table gives the number of cases where a SNP is present either at the miRNA binding site (TSNP), upstream (USNP) or downstream (DSNP) of the miRNA binding site in the 3' UTR of the target transcript. The polymorphism may alter the intramolecular structure at the miRNA binding site either significantly (where 30% or more bases change their binding modalities to create a structured region closed (Gain) or a closed region getting opened up (Loss) resulting in gain or loss of the miRNA binding respectively), moderately if less than 30% bases alter their binding modalities or no effect on local intramolecular structure at all with the wild and polymorphic allele.

## Utility and Discussion

Data pertaining to validated miRNA-target pairs allows further studies on the the effect of polymorphism, not just at the target site of miRNA binding, but also in the region around them. Two miRNAs (hsa-miR-15a and hsa-miR-16) are experimentally demonstrated to target the BCL2 transcript. The deletion of this miRNA cluster in B-cell lymphoma has been implicated in B-cell lymphoma [29]. We notice that a polymorphism 172 bases upstream of the target site for the miRNAs (rs4987856) can alter the highly accessible structure to an inaccessible site (Figure 1b) for the miRNAs hsa-miR-15a and hsa-miR-16. This structural alteration might not enable miRNA interaction to the transcript harboring the polymorphic allele, mimicking the effect that of deleted miRNAs as in case of B-cell lymphoma patients.

We further analyzed the selection pressure on those SNPs which alter miRNA binding due to the structural effects. The integrated haplotype score (iHS) is a standardized measure of long range haplotype for a particular SNP in a given population. The same approach was used in a recent paper which performed a genome-wide scan of SNPs at miRNA binding sites [16]. The iHS values for all SNPs available from HapMap phase 2 data in three population – ASI (Chinese and Japanese), YRI and CEU) were obtained from Haplotter website . Data for only those SNPs which have minor allele frequency (MAF) > 5% were available. We found that very few (only 1–2%) of the SNPs that change the miRNA accessibility were prone to either positive or negative selection (iHS < -2 or iHS > 2, respectively). The SNPs rs140074 (in the PATZ1 3'UTR) and rs11848279 (in the NFATC4 3'UTR) indicate negative selection (in Yoruban and Caucasian population) and positive selection (in Yoruban and Caucasian population) respectively.

It is appreciated that secondary structures are common in the UTRs of the transcripts. It is also clear from several studies that interaction of miRNAs to the target site is governed to a large extent by the structural accessibility to these sites. Since polymorphisms can alter the structure of these regions, we propose that variations in the 3' UTRs, even if farther away from the target site can alter the miRNA binding and hence would contribute to this additional layer of regulation. Stable structural motifs in the target sites would be inaccessible for miRNAs thereby constraining miRNA mediated regulation. The large activation energy involved in destabilizing the mRNA secondary structure would render interactions within a secondary structure forming region kinetically non-feasible even when thermodynamically viable. Others and we have previously devised approaches to incorporate the structural architecture of target regions into miRNA target prediction. Comparing the free energy difference of the intramolecular interaction with that of the interaction with the miRNAs, it is possible to identify thermodynamically feasible interactions of miRNA with the target site. Although currently available reports suggest direct involvement of SNPs in the miRNA target site whereby a nucleotide that interacts with the miRNA itself changes altering the intermolecular energy (Minimal Free Energy of the complex), we notice that variations away from the target site (the target region) can also affect miRNA accessibility. The loss of miR-24 targeting DHFR transcript due to a T-allele 14 nt downstream of the predicted target site was demonstrated to reduce the half life of the transcript [18]. The authors propose that the region 14 nt downstream of the target site is important in the binding of the Ago proteins. However, we find that there is a significant change in the structural conformation of the UTR of DHFR. While the UTR exists in a highly structured form with a 'T' allele, the UTR which harbors a 'C' is highly unstructured. This would be a cause for the increase miRNA binding affinity to the target region of the UTR with the 'C' allele (Figure 1c).

It is difficult for individual investigators to look at the overall complexity in the context of genetic variation. Hence the dataset presented would be of immense value for researchers. In this paper, we have analyzed and catalogued polymorphisms that would make some individual specific genes more susceptible (or otherwise) to miRNA mediated regulation due to such changes. As demonstrated in the case of the validated miR-15a/miR-16 target site in BCL2 gene, a stretch of intramolecular bond formation at the interacting site of the miRNA in the UTR might lead to loss of miRNA binding. It remains open for experimentalists to validate such interesting possibilities and study various complexities involved in miRNA-target interactions. It would be worthwhile to identify polymorphisms with high polymorphic allele frequencies that have an effect on miRNA accessibility. Linking the functional role of the target gene and known effects of the miRNA binding, investigators can detect novel regulatory components that are prevalent in certain population which make them susceptible or otherwise, to miRNA mediated PTGS. Such a resource would enable researchers address questions like the role of regulatory SNPs in the 3' UTRs and population specific regulatory modulations.

As validation and experimental confirmation of miRNA-target interactions increase, we aim to keep the database regularly updated. In the next version, we also plan to include a graphical representation of the intramolecular structural changes. Although most users would require the data pertaining to a specific gene or a miRNA, we plan to incorporate a representation of the polymorphism and target region as an interactive map in the forthcoming improvement.

## Conclusion

There have been several studies which have proven the detrimental effects of polymorphisms at the miRNA target site. Various structural analyses have also shown that accessibility of the miRNAs at the target site is an important factor that governs the miRNA mediated regulation. Polymorphisms that can alter the secondary structure at the miRNA binding region can thus have a significant role in controlling the accessibility of the miRNAs.

Through the genome-wide miRNA prediction performed here, we have collated the information of all validated SNPs that can affect the secondary structure of the miRNA binding regions, at varying degrees. Such a resource would enable researchers address questions like the role of regulatory SNPs in the 3' UTRs and population specific regulatory modulations. The true significance of the principle can be realized when the effect of these polymorphisms is studied at population level or in case-control disease samples. These would allow conclusive classification of SNPs as detrimental to miRNA binding or not, based on the information provided. We hope the database provides the necessary support for such high-throughput and thorough analysis

## Availability and Requirements

The dbSMR database is freely available to all academic and users and is accessible through the URL: 

## Authors' contributions

MH, VS and SKB conceived the hypothesis. MH generated the data, developed the database and wrote the manuscript. VS maintains the server. All authors read and approved the final manuscript.

## References

[B1] He L, Hannon GJ (2004). MicroRNAs: small RNAs with a big role in gene regulation. Nat Rev Genet.

[B2] Scaria V, Hariharan M, Pillai B, Maiti S, Brahmachari SK (2007). Host-virus genome interactions: macro roles for microRNAs. Cell Microbiol.

[B3] Pillai RS, Bhattacharyya SN, Filipowicz W (2007). Repression of protein synthesis by miRNAs: how many mechanisms?. Trends Cell Biol.

[B4] Lai EC (2004). Predicting and validating microRNA targets. Genome Biol.

[B5] Brennecke J, Stark A, Russell RB, Cohen SM (2005). Principles of microRNA-target recognition. PLoS Biol.

[B6] Doench JG, Sharp PA (2004). Specificity of microRNA target selection in translational repression. Genes Dev.

[B7] Robins H, Li Y, Padgett RW (2005). Incorporating structure to predict microRNA targets. Proc Natl Acad Sci USA.

[B8] Zhao Y, Samal E, Srivastava D (2005). Serum response factor regulates a muscle-specific microRNA that targets Hand2 during cardiogenesis. Nature.

[B9] Kertesz M, Iovino N, Unnerstall U, Gaul U, Segal E (2007). The role of site accessibility in microRNA target recognition. Nat Genet.

[B10] Long D, Lee R, Williams P, Chan CY, Ambros V, Ding Y (2007). Potent effect of target structure on microRNA function. Nat Struct Mol Biol.

[B11] Thadani R, Tammi MT (2006). MicroTar: predicting microRNA targets from RNA duplexes. BMC Bioinformatics.

[B12] Muckstein U, Tafer H, Hackermuller J, Bernhart SH, Stadler PF, Hofacker IL (2006). Thermodynamics of RNA-RNA binding. Bioinformatics.

[B13] http://miracle.igib.res.in/miracle.

[B14] Abelson JF, Kwan KY, O'Roak BJ, Baek DY, Stillman AA, Morgan TM, Mathews CA, Pauls DL, Rasin MR, Gunel M (2005). Sequence variants in SLITRK1 are associated with Tourette's syndrome. Science.

[B15] Clop A, Marcq F, Takeda H, Pirottin D, Tordoir X, Bibe B, Bouix J, Caiment F, Elsen JM, Eychenne F (2006). A mutation creating a potential illegitimate microRNA target site in the myostatin gene affects muscularity in sheep. Nat Genet.

[B16] Saunders MA, Liang H, Li WH (2007). Human polymorphism at microRNAs and microRNA target sites. Proc Natl Acad Sci USA.

[B17] Yu Z, Li Z, Jolicoeur N, Zhang L, Fortin Y, Wang E, Wu M, Shen SH (2007). Aberrant allele frequencies of the SNPs located in microRNA target sites are potentially associated with human cancers. Nucleic Acids Res.

[B18] Martin MM, Buckenberger JA, Jiang J, Malana GE, Nuovo GJ, Chotani M, Feldman DS, Schmittgen TD, Elton TS (2007). The human angiotensin II type 1 receptor +1166 A/C polymorphism attenuates microrna-155 binding. J Biol Chem.

[B19] Mishra PJ, Humeniuk R, Mishra PJ, Longo-Sorbello GS, Banerjee D, Bertino JR (2007). A miR-24 microRNA binding-site polymorphism in dihydrofolate reductase gene leads to methotrexate resistance. Proc Natl Acad Sci USA.

[B20] Griffiths-Jones S, Saini HK, van Dongen S, Enright AJ (2008). miRBase: tools for microRNA genomics. Nucleic Acids Res.

[B21] Flicek P (2008). Ensembl 2008. Nucleic Acids Res.

[B22] Rajewsky N (2006). microRNA target predictions in animals. Nat Genet.

[B23] Enright AJ, John B, Gaul U, Tuschl T, Sander C, Marks DS (2003). MicroRNA targets in Drosophila. Genome Biol.

[B24] Rehmsmeier M, Steffen P, Hochsmann M, Giegerich R (2004). Fast and effective prediction of microRNA/target duplexes. RNA.

[B25] Lewis BP, Shih IH, Jones-Rhoades MW, Bartel DP, Burge CB (2003). Prediction of mammalian microRNA targets. Cell.

[B26] Hofacker W, Fontana PF, Stadler S, Bonhoeffer M, Tacker P, Schuster (1994). Fast Folding and Comparison of RNA Secondary Structures. Monatshefte f Chemie.

[B27] Gardner PP, Giegerich R (2004). A comprehensive comparison of comparative RNA structure prediction approaches. BMC Bioinformatics.

[B28] Sethupathy P, Corda B, Hatzigeorgiou AG (2006). TarBase: A comprehensive database of experimentally supported animal microRNA targets. RNA.

[B29] Cimmino A (2005). miR-15 and miR-16 induce apoptosis by targeting BCL2. Proc Natl Acad Sci USA.

